# Advanced Anticorrosive Graphene Oxide-Doped Organic-Inorganic Hybrid Nanocomposite Coating Derived from *Leucaena leucocephala* Oil

**DOI:** 10.3390/polym15224390

**Published:** 2023-11-12

**Authors:** Wejdan Al-otaibi, Naser M. Alandis, Yasser M. Al-Mohammad, Manawwer Alam

**Affiliations:** Department of Chemistry, College of Science, King Saud University, P.O. Box 2455, Riyadh 11451, Saudi Arabia; wejdanotaiby@gmail.com (W.A.-o.); nandis@ksu.edu.sa (N.M.A.);

**Keywords:** bio-resource, polyurethanamide, organic-inorganic hybrid, nanocomposite, coating, corrosion inhibition

## Abstract

Metal corrosion poses a substantial economic challenge in a technologically advanced world. In this study, novel environmentally friendly anticorrosive graphene oxide (GO)-doped organic-inorganic hybrid polyurethane (LFAOIH@GO-PU) nanocomposite coatings were developed from *Leucaena leucocephala* oil (LLO). The formulation was produced by the amidation reaction of LLO to form diol fatty amide followed by the reaction of tetraethoxysilane (TEOS) and a dispersion of GO_x_ (X = 0.25, 0.50, and 0.75 wt%) along with the reaction of isophorane diisocyanate (IPDI) (25–40 wt%) to form LFAOIH@GO_x_-PU_35_ nanocomposites. The synthesized materials were characterized by Fourier transform infrared spectroscopy (FTIR); ^1^H, ^13^C, and ^29^Si nuclear magnetic resonance; and X-ray photoelectron spectroscopy. A detailed examination of LFAOIH@GO_0.5_-PU_35_ morphology was conducted using X-ray diffraction, scanning electron microscopy, energy-dispersive X-ray spectroscopy, and transmission electron microscopy. These studies revealed distinctive surface roughness features along with a contact angle of around 88 G.U preserving their structural integrity at temperatures of up to 235 °C with minimal loading of GO. Additionally, improved mechanical properties, including scratch hardness (3 kg), pencil hardness (5H), impact resistance, bending, gloss value (79), crosshatch adhesion, and thickness were evaluated with the dispersion of GO. Electrochemical corrosion studies, involving Nyquist, Bode, and Tafel plots, provided clear evidence of the outstanding anticorrosion performance of the coatings.

## 1. Introduction

Corrosion is a process that diminishes the strength and integrity of metallic materials [1]. It can be initiated by both natural processes and human activities and is influenced by a range of factors, including humidity, gaseous surroundings, electrolytes, temperature, and pH variations [2,3]. The economic consequences associated with corrosion are notably substantial, and the corrosion protection coating market is anticipated to witness robust expansion, progressing from an initial valuation of USD 10.4 billion in 2023 to a projected sum of USD 12.4 billion by the year 2028 [4]. This expense increases in parallel with technological advancement, affecting the worldwide economy and human welfare. The utilization of polymeric coatings emerges as a prime strategy to protect metallic materials against corrosive environments. Amongst the various polymeric materials employed as coatings, organic-inorganic hybrid (OIH) coatings stand out for their extensive utilization for advanced applications [5,6]. The utilization of OIH materials has garnered considerable attention in the research field due to their capacity to systematically amalgamate and regulate properties stemming from both organic and inorganic components. These hybrid materials are distinguished by their exceptional mechanical, thermal, cryogenic, and dynamic attributes, rendering them highly advantageous for a wide spectrum of challenging applications [5]. Significantly, these applications encompass the development of coatings for automotive, aircraft, aerospace, optically active films, contact lenses, packaging materials, abrasion-resistant paints, and protective coatings [7].

The escalating environmental apprehensions encompassing issues like toxicity, high cost, gas flaring, and industrial/domestic waste, coupled with the volatile and unpredictable pricing dynamics of petroleum-derived feedstocks, bear significant emphasis. The global economy’s dependence on this exhaustible, environmentally non-benign resource underscores the necessity of replacing this indispensable energy and feedstock source with sustainable, eco-friendly materials [8]. Hence, in the development of OIH materials, the substitution of petroleum-derived organic components with bio-based alternatives such as polysaccharides, cellulose, proteins, cashew nut shell liquid, and vegetable seed oils (VSOs) offers advantageous prospects [9]. VSOs have gained notable recognition as a promising source of bio-polyols, largely due to their abundant accessibility, uncomplicated processing, and inherent chemical versatility [10]. Organic domains derived from vegetable oils exhibit significant promise as viable substitutes of petroleum products resulting in improved performance attributes with substantial ecological and economic implications. Various materials obtained from VSOs, such as soybean, rapeseed, castor, and *Leucaena leucocephala* (*LL*), have been meticulously designed to cater to specific application requirements [9]. Among different VSOs, *LL oil* (*LLO*), produced from LL, a member of the Fabaceae family, is an agro-industrial product with diverse applications in the realm of bioenergy. LLO derivatives like polyetheramide, polyesteramide, and polyurethane have been utilized in the field of anticorrosive coatings. But there is a further need to improve their mechanical and anticorrosive performance.

Integrating graphene oxide (GO) into polymers as nanocomposites offers notable benefits, including improved flexibility and electrical conductivity through its enhanced electron mobility and surface area, as shown by graphene’s versatile use in various applications [11]. In recent times, there has been a surge of interest in GO-doped OIH materials among researchers due to their strong thermal and electrical stability and mechanical strength, with graphene emerging as a pivotal dispersion component. Al Rashed et al. [12] synthesized polyurethane/polysiloxane OIH hybrid coatings filled with GO, which revealed good mechanical and optical properties. The results demonstrated that GO, along with isocyanate treatment, reduced oxygen and moisture penetration in the coatings. The effect of GO facilitated polysiloxane condensation on their surfaces, enhancing corrosion protection on aluminum alloy surfaces. Harb et al. [13] designed GO-doped OIH coatings for protecting steel surfaces from corrosion by benzoyl peroxide-induced polymerization of methyl methacrylate covalently bonded through 3-(trimethoxysilyl)propyl methacrylate to silica domains formed by the hydrolytic condensation of tetraethoxysilane. Ahmad et al. [5] fabricated OIH coatings composed of polyurethane fatty amide and silica components under ambient conditions. The synthesized material had high physicomechanical, chemical, and thermal stability and was produced with the objective of addressing corrosion issues in mild steel. A literature survey reveals that no work has been reported on the proposed research work.

The aim of the current study is to achieve a sustainable and environmentally friendly synthesis of GO-doped *LLO*-based organic-inorganic hybrid (LFAOIH) polyurethane (PU) material (LFAOIH@GO-PU) for advanced anticorrosive application. TheLFAOIH@GO-PU formulation was produced in two simple steps: (i) preparation of *LLO*-based diol fatty amide (LLFAD) and (ii) reaction of freshly prepared LLFAD with tetraethoxy silane (TEOS) to form an organic-inorganic hybrid (LFAOIH), followed by in situ dispersion of GO (different percentages) to form LFAOIH @GO, followed by the reaction of isophorane diisocyanate (IPDI) to form ambient cured LFAOIH@GO-PU coating material. The present work explores the utility of synthesized nanocomposite material in mechanically robust chemically resistant and anticorrosive coatings. Notably, the study pioneers the application of Electrochemical Impedance Spectroscopy (EIS) to elucidate the corrosion protection in the presence of 3.5% NaCl, employing Nyquist and Bode plots over for 12 days along with the potentiodynamic (Tafel) analysis. The investigation also encompasses the structural validation through Fourier transform infrared (FTIR), ^1^H/^13^C/^29^Si Nuclear magnetic resonance (NMR) and X-ray photoelectron spectroscopy (XPS). Additionally, the morphology is scrutinized using X-ray diffraction (XRD), scanning electron microscopy (SEM), and transmission electron microscopy (TEM). The thermal stability aspect is addressed through thermogravimetric and differential scanning calorimetry analysis (TGA/DTG and DSC). Furthermore, the introduced one-pot synthesis process from LLFAD contributes significantly to the fabrication of eco-friendly polymeric protective coatings.

## 2. Experimental

### 2.1. Materials and Methods

The study involved the extraction of *Leucaena leucocephala* seed oil from mature legumes collected at the University Campus of King Saud University. The seeds were subsequently ground into a powdered form, and the oil was then extracted from the powdered seeds utilizing a Soxhlet apparatus with petroleum ether as the chosen solvent. The elimination of ether was achieved through the use of a vacuum rotary evaporator [14]. Diethanolamine was procured from AppliChem GmbH in Darmstadt, Germany, while sodium metal, sodium chloride, and toluene were acquired from Winlab in the United Kingdom. Additionally, the reagents TEOS (98%) and IPDI (98%) were sourced from Acros Organics in NJ, USA. Notably. GO was obtained from Grafen Chemical Industries Co in Ankara, Turkey. Diethyl ether, methanol, and dibutyltin dilaurate were purchased from Sigma-Aldrich in St. Louis, NJ, USA.

### 2.2. Synthesis of Leucaena leucocephala Fattyamide Diol (LLFAD)

LLFAD was prepared as per the reported method [5].

### 2.3. Synthesis of LFAOIH and Its PU (LFAOIH-PU_30-40_)

Freshly prepared LLFAD (14 g, 0.04 mole) was introduced into a three-necked conical flask equipped with essential apparatuses, including a dropping funnel, thermometer, and condenser. Sequentially, 8.32 g (0.04 moles) of TEOS was added in a controlled manner over a 30 min interval under continuous agitation at room temperature. Upon complete incorporation of TEOS, the reaction mixture was subjected to an elevated temperature of 80 ± 5 °C for 1 h. The contents were further heated at 120 ± 5 °C for 1 h to complete the reaction. The progression of the reaction was diligently monitored via appearance of contents (conversion of fogginess to clear) and FTIR. Subsequently, the reaction mixture underwent gradual cooling to ambient temperature while maintained in continuous agitation. Within the same experimental context, incremental additions of IPDI were meticulously introduced, each at varying proportions (30, 35, and 40% with regard to LLFAD) over 30 min duration with a drop of DBTL and maintained the temperature at 60 °C for 3 h. A minute volume (8–12%) of toluene was incorporated to sustain the desired viscosity. The trajectory of the reaction was monitored by FTIR. The resulting LFAOIH-PU products were denoted as LFAOIH-PU_30_, LFAOIH-PU_35_, and LFAOIH-PU_40_, the subscript numeral indicating the % of IPDI.

### 2.4. Synthesis of LFAOIH@GO and Their PU Nanocomposite (LFAOIH@GO_x_-PU_35_)

LFAOIH was synthesized as the aforementioned setup with an equimolar amount of LLFAD and TEOS (0.04 moles). GO_X_ (X = 0.25%, 0.50% and 0.75% w.r.t. LLFAD), initially dispersed (by sonication for 10 min) in minimal amounts of toluene, was added in the same pot at the same temperature. The temperature was maintained for 1 h. The reaction progress was tracked by using viscosity and FTIR. Afterward, the mixture was slowly cooled with continuous agitation. In the same vessel, an optimized 35% IPDI, along with a drop of DBTL, was added over 30 min and at 60 °C for 3 h. A small volume of toluene (8–12%) was used to maintain the desired viscosity. The trajectory of the reaction was checked by viscosity and FTIR analysis. The resulting products were denoted as LFAOIH@GO_0.25_-PU_35_, LFAOIH@GO_0.5_-PU_35_, and LFAOIH@GO_0.75_-PU_35_.

### 2.5. Characterizations

Various spectroscopic techniques were employed to characterize the prepared samples. FTIR measurements were conducted using FTIR spectrophotometer; Spectrum 100, Perkin Elmer Cetus Instrument, Norwalk, CT, USA spanning a range of 4000 to 400 cm^−1^ at room temperature (28–30 °C) with a resolution of 4 cm^−1^. Nuclear Magnetic Resonance (NMR) spectra, specifically ^1^H, ^13^C, and ^29^Si NMR, were obtained using a Jeol DPX400 MHz instrument, Tokyo, Japan. Deuterated dimethyl sulfoxide (DMSO) served as the solvent, while tetramethyl silane was used as an internal standard for calibration. The sample was analyzed by X-ray photoelectron spectroscopy (XPS) by using a JPS-9030; JEOL, Japan MgKα (1253.6 eV). The solubility assessment of the prepared sample involved dissolving a 20 mg sample in 10 mL of both polar and nonpolar solvents (dimethyl sulfoxide, pyridine, benzene, toluene, ethyl-methyl ketone, dichloromethane, butanol, chloroform, diethyl ether, benzyl alcohol, carbon tetrachloride, N,N-dimethyl formamide, isoamyl alcohol, petroleum ether, butan-1-ol, 1-hexanol, cyclohexene, furan, propanol, 1,4-dioxane, ethanol, acetone, and ethyl acetate). The resulting solution was allowed to stand undisturbed for a duration of 24 h. Various physico-mechanical tests were conducted, including thickness measurements using ASTM D1186-B, Elcometer Model 345 from Elcometer Instrument Ltd. in Manchester, UK, scratch hardness following BS 3900 standards, pencil hardness in accordance with ASTM D3363-05, crosshatch testing following ASTM D3359-02, impact testing as per IS 101:1988, bend testing based on ASTM D3281-84, and gloss measurement using a Gloss meter Model KSJ MG6-F1 from KSJ Photoelectrical Instruments Co., Ltd. in Quanzhou, China. To investigate the composition and morphology of the film, a scanning electron microscope (SEM), namely the JSM 7600F by JEOL in Japan, was utilized. For elemental analysis, Energy Dispersive X-ray spectroscopy (EDX) coupled to the SEM was employed. To facilitate observation under the microscope, the sample was sputter-coated with Pt and the microscope operated at 15 kV. Transmission electron microscopy (TEM) was conducted using a JEM-2100F transmission electron microscope by JEOL, Japan. For checking the wettability of the surface, a drop of double distilled water was deposited on the surface of the coating and photographed with contact angle measurements (CAM200 Attention goniometer). Electrochemical impedance spectroscopy (EIS) was employed to assess the corrosion resistance of the prepared sample in a 3.5% NaCl solution. This analysis was conducted using a three-electrode flat glass cell, alongside an Autolab potentiostat/galvanostat (PGSTAT204-FRA32) and NOVA 2.1 software. The equipment used for this purpose was Metrohom Autolab B.V., located at Kanaalweg 29-G, 3526 KM, Utrecht, Netherlands. The experimental setup involved three electrodes: (I) Ag/AgCl utilized as a reference electrode, (II) platinum serving as an auxiliary electrode, and (III) the test specimen functioning as the working electrode. The solution was applied to a 1.0 cm^2^ area of the working electrode, adhering to the ASTM G59-97 standard. The purpose was to gauge the performance of the coated surface in terms of its impedance behavior against corrosion in the specified environment. The electrochemical parameters such as corrosion potential (Ecorr) and current density (Icorr) were calculated from Tafel extrapolation method and the corrosion inhibition efficiency can be calculated form Equation (1)

(1) $$IE\%=\frac{corrosion\;current\;density\;of\;\left(bare\;metal-coated\;surface\right)}{corrosion\;current\;density\;of\;coated\;surface}\times 100$$

## 3. Results and Discussion

**Scheme 1:**
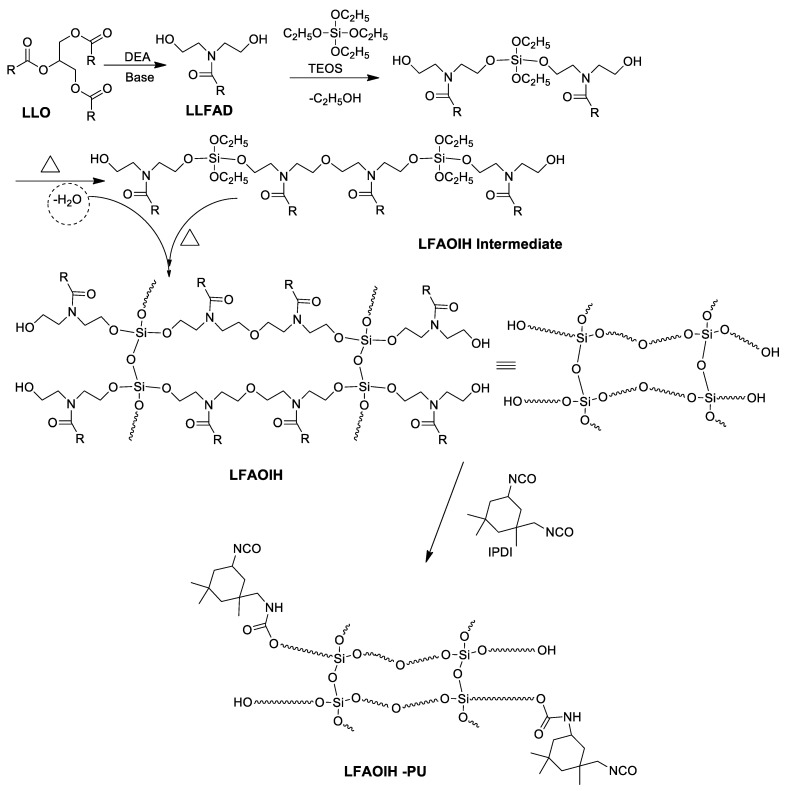
Synthesis of LFAOIH-PU.

**Scheme 2:**
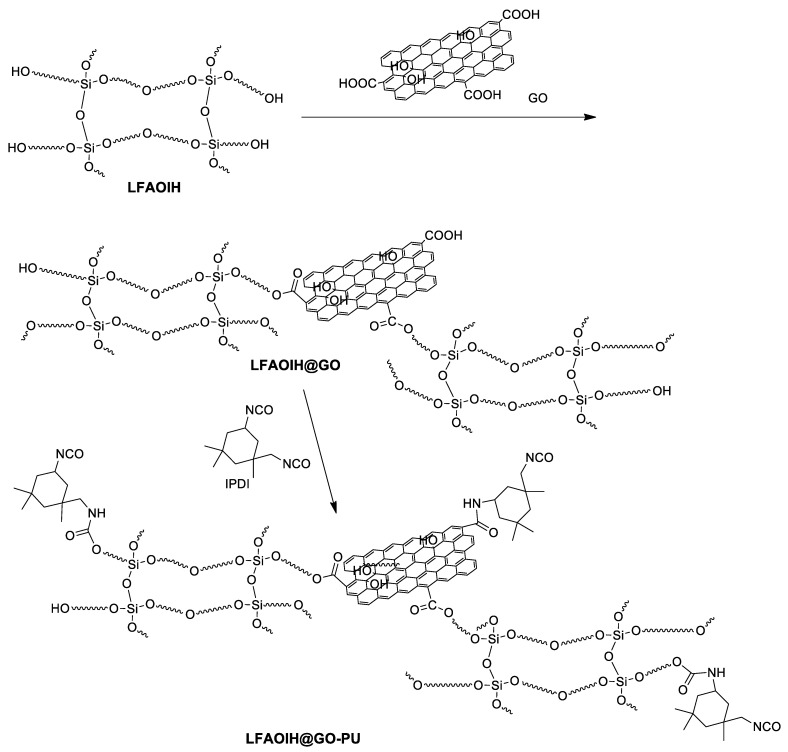
Synthesis of LFAOIH@GO-PU.

Scheme 1 and Scheme 2 show the synthesis of LFAOIH-PU_35_ and LFAOIH@GO_x_-PU_35_ nanocomposite material from LLO. It reveals the formulation carried out in two different steps. The first step of reaction involves the conversion of LLO into LLFAD by the base catalyzed reaction of LLO with diethanolamine to form LLFAD. Then, the purified LLFAD reacted with the equimolar amount of TEOS to form LFAOIH at 100 ± 5 °C. When TEOS was introduced to LLFAD, it was noticed that the reaction mixture became cloudy at 80 ± 5 °C; however, this cloudiness vanished after 1 h of heating at 120 ± 5 °C. This is connected to the chemical reaction between the hydroxyl groups of LLFAD and the alkoxy group of TEOS, which generates O-Si-O bonds as a result of the reaction [5]. After that dispersion of GOx (X = 0.25%, 0.50% and 0.75% w.r.t. LLFAD) followed by the reaction of the content with an optimized percentage of IPDI (35%) at 60 ± 5 °C temperature to form the crosslinked material for coating. IPDI crosslinked the materials through the reaction of –NCO with the residual functionality of LFAOIH@GO_0.5_ to form LFAOIH@GO_0.5_-PU_35_. The solubility behavior of the LFAOIH@GO_0.5_-PU_35_ nanocomposite material is influenced by the polarities, functional groups, and molecular structures of both the nanocomposite itself and the solvents. Solubility in dimethyl sulfoxide, pyridine, benzene, toluene, ethyl-methyl ketone, dichloromethane, butanol, and chloroform may be due to the presence of compatible functional groups and molecular arrangements [15,16]. The interactions between these components likely facilitate dissolution. The insolubility in diethyl ether, benzyl alcohol, carbon tetrachloride, isoamyl alcohol, petroleum ether, butan-1-ol, 1-hexanol, and cyclohexane may result from differences in polarity, hydrogen bonding capabilities, and molecular sizes between both the nanocomposite and these solvents. These factors hinder the establishment of strong enough interactions to enable dissolution [9]. The materials were found to be partially soluble in cyclohexene, furan, propanol, 1,4-dioxane, ethanol, acetone, and ethyl acetate, which may be due to the compatibility between the nanocomposite and these solvents, allowing for some degree of dissolution due to similar chemical properties.

### 3.1. Spectral Analysis

#### 3.1.1. FTIR Analysis

**Figure 1:**
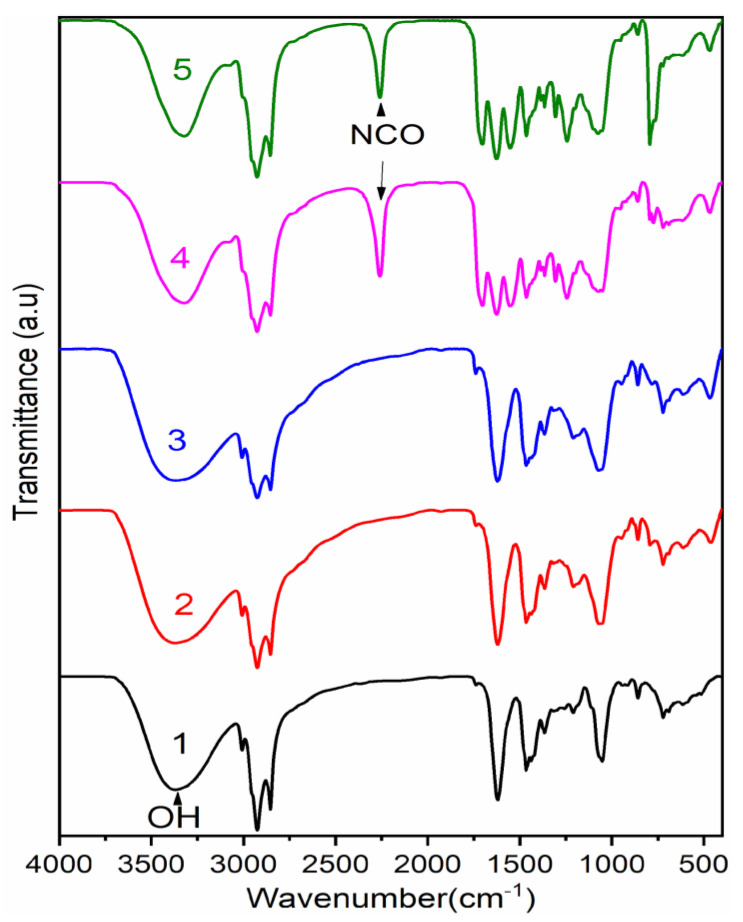
FTIR spectra of (**1**) LLFAD, (**2**) LFAOIH, (**3**) LFAOIH@GO, (**4**) LFAOIH−PU_35_ and (**5**) LFAOIH@GO_0.5_-PU_35_.

The formation of LFAOIH-PU_35_ and LFAOIH@GO_0.5_-PU_35_ was confirmed through FTIR spectrum analysis. To validate this confirmation, a comparison was carried out by analyzing the FTIR spectra of LLFAD, LFAOIH-PU_35_ and LFAOIH@GO_0.5_-PU_35_, as shown in Figure 1. The characteristics peaks are as:

LLFAD: In the FTIR analysis of LLFAD, distinct absorption bands were observed corresponding to specific functional groups and molecular vibrations [Figure 1(1)]. These include an absorption band at 3359 cm^−1^ attributed to hydroxyl groups (-OH), indicating the presence of alcohols. The band at 3008 cm^−1^ signifies the presence of C=C double bonds. Bands at 2853 cm^−1^ and 2924 cm^−1^ correspond to alkyl stretching vibrations, indicating the presence of methyl (-CH_3_) and methylene (-CH_2_) groups [14]. The absorption band at 1618 cm^−1^ is indicative of the amide carbonyl group (C=O) stretching vibration. The region between 1466 cm^−1^ and 1384 cm^−1^ represents the bending vibrations of methyl and methylene groups (-CH_3_, -CH_2_). The bands at 1210 cm^−1^ and 1052 cm^−1^ are characteristic of the ether linkage (-(C=O)-O-C) vibration [16], and the presence of -C-O- bonds, respectively [14].

LFAOIH: In the FTIR analysis of LFAOIH, the observed absorption bands are consistent with those in LLFAD, indicating the presence of similar functional groups. Notably, additional peaks corresponding to silicon oxide interactions are also evident [14]. Furthermore, distinctive peaks associated with silicon oxide interactions emerge, encompassing a symmetrical stretch at 614 cm^−1^ (Si-O-Si), an asymmetrical stretch at 1069 cm^−1^ (Si-O-Si), and a bending vibration at 461 cm^−1^ (Si-O-Si) [5]. These absorptions signify the involvement of silicon oxide moieties within the LLFAD-TEOS compound.

LFAOIH-PU: The hydroxyl group (-OH) peak, previously at 3368 cm^−1^, shifts to 3323 cm^−1^, which may be due to hydrogen bonding [Figure 1]. The peak appears at 1704 cm^−1^ related to the urethane carbonyl{-O-C(=O)-NH-} [5,14]. Other peaks include 1626 cm^−1^, 793 cm^−1^ (ArC=C- bending), 468 cm^−1^ (Si-O-Si bending), and 1074 cm^−1^ (asymmetrical Si-O-Si stretch) [5], with notable peaks at 2259 cm^−1^ (-NCO group). This analysis provides insights into molecular interactions and the polyurethane’s structure.

LFAOIH@GO_x_: The detection of GO incorporation is not primarily determined by the presence of GO’s absorption bands, as GO encompasses numerous -O-containing functional groups that can overlap with those present in the LLFAD (Figure 1). Instead, the indication of GO’s presence is derived from shifts in absorption values. LFAOIH@GO_0.5_ has all the characteristic peaks as found in LFAOIH along with broadening of the peak at 3368 due to hydrogen bonding and additional peaks at 1738 cm^−1^, which is due to ester C=O that formed by the reaction of the hydroxyl group of LFAOIH with -COOH group GO [16,17]. The detection of GO’s presence may not be evident solely through the observation of absorption bands associated with GO because it possesses numerous -O- functional groups that often overlap with functional groups found. Instead, one can identify the presence of GO by noting changes in the absorption values of specific functional groups like -OH, >C=O ester and >C=O amide in comparison to those observed in virgin LFAOIH. These alterations in absorption values are attributed to the interactions occurring between LFAOIH and GO [18,19,20].

LFAOIH@GO_0.5_-PU_35_: The emergence of an additional peak at 2259 cm^−1^ and 1704 cm^−1^ (overlap with >C=O ester peak of LFAOIH@GO_x_) can be attributed to the presence of the urethane linkage formed between the isocyanate (-NCO) and hydroxyl (-OH) groups during the process of polymerization along with the same peaks found in LFAOIH@GO_0.5_, which confirmed LFAOIH@GO_0.5_-PU_35_ formulation (Figure 1) [16,17].

The broad hump in the FTIR spectrum in the 3200–3500 cm^−1^ region is a distinctive indication of the presence of hydrogen bonding. It results from the broadening of the O-H stretching vibration peak due to the dynamic and variable nature of hydrogen bond interactions within the sample [18]. The comparative FTIR data of LFAOIH-PU_35_ and LFAOIH@GO_0.5_-PU_35_ [Figure 1] indicate that the peaks of LFAOIH@GO_0.5_-PU_35_ shows a broad hump as compared to LFAOIH-PU_35,_ which revealed the characteristic of hydrogen bonding and indicates that multiple O-H groups in the sample are engaged in hydrogen bond interactions. Hydrogen bonding exerts a discernible influence on the enhancement of mechanical properties, including scratch hardness, pencil hardness, bending and gloss values [19]. Robust hydrogen bonding fosters enhanced adhesion between coatings and substrates, and bolsters material toughness. It also contributes to resistance against wear and abrasion and augments ductility, mitigating the susceptibility to deformation-induced cracking [20].

#### 3.1.2. NMR Analysis

**Figure 2:**
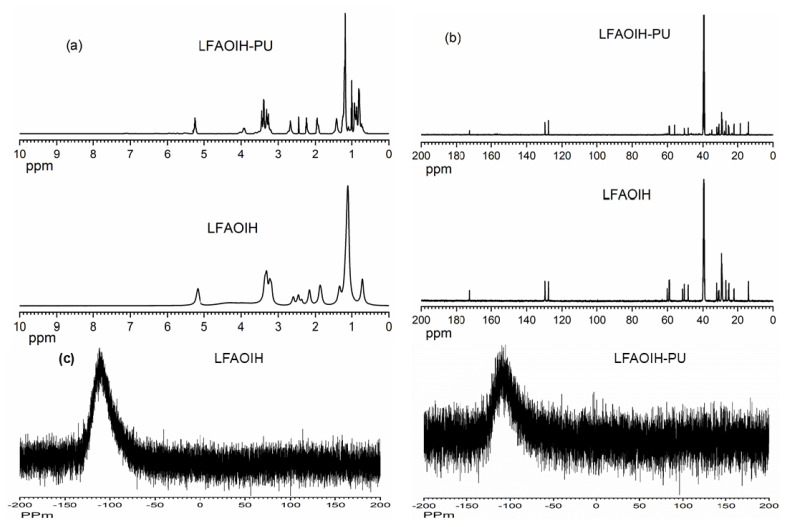
(**a**) ^1^H, (**b**) ^13^C and (**c**) ^29^Si NMR spectra of LFAOIH and LFAOIH-PU, respectively.

^1^H NMR: In the ^1^H NMR spectrum of LFAOIH, distinct peaks were observed (Figure 2a), such as 0.715 ppm for -C**H**_3_ groups, 1.08–1.21 ppm for -C**H**_2_ groups, and 1.329 ppm for >N-CO-CH_2_-C**H**_2_ moieties. Other notable peaks included 1.87 ppm (-CH_2_-C**H**_2_-CH=), 2.16 ppm (>N-CO-C**H**_2_-), 2.59 ppm (=CH-C**H**_2_-N), and broad 3.5 ppm (-C**H**-O-Si-), as well as 5.23 ppm (-O**H**) [21], whereas in the formulation of LFAOIH-PU_35_, the same characteristic peaks were observed, with a sharper and quadruple 3.5 ppm (-C**H**-O-Si-) peak due to its involvement in the reaction. Additionally, a minor peak at ~4 ppm, attributed to (-O-C**H**_2_-) from the incorporation of IPDI, and a sharp peak around 5.2 ppm confirmed polymerization via IPDI insertion.

^13^C NMR: In the ^13^C NMR spectrum of LFAOIH, specific resonance peaks were identified, including 13.96 ppm (attributed to -**C**H_3_), a range spanning 21.14–33.90 ppm (associated with the **C**H_2_ chain), and a prominent peak at 40.1 ppm indicative of dimethyl sulfoxide (DMSO) (Figure 2b) [21]. Additionally, resonances were observed within the range of 49.01–59.12 ppm, corresponding to the >N-CO-**C**H_2_**C**H_2_- moiety. Further distinctive signals were detected at 129–130.01 ppm (-**C**H=CH-) and 171 ppm (-**C**H-O-Si-) [22]. In the LFAOIH-PU_35_ formulation, all these characteristic peaks were retained; however, a noteworthy observation was the diminished intensity of the peak around 171 ppm. This attenuation suggests a plausible interaction involving this carbon in subsequent reactions with IPDI and the formulation of LFAOIH-PU_35_.

^29^Si NMR: In the ^29^Si NMR spectrum, a sharp chemical shift around −105 ppm has been found, which confirmed the presence of Si-O-Si atoms (Q4species) bonded to four oxygen atoms in siloxane (-Si-O-) within the compound (Figure 2c) [22]. In the case of LFAOIH, the peak around −100 is found to be sharp whereas in the exact peak it was found to be a little broad. The broadening of the same (−105 ppm) peak in the spectrum when reacting with IPDI is a result of the chemical changes and increased structural complexity causing dynamic exchange between silicon species and yielding a highly crosslinked structure of LFAOIH-PU_35_ [22].

#### 3.1.3. XPS Analysis

**Figure 3:**
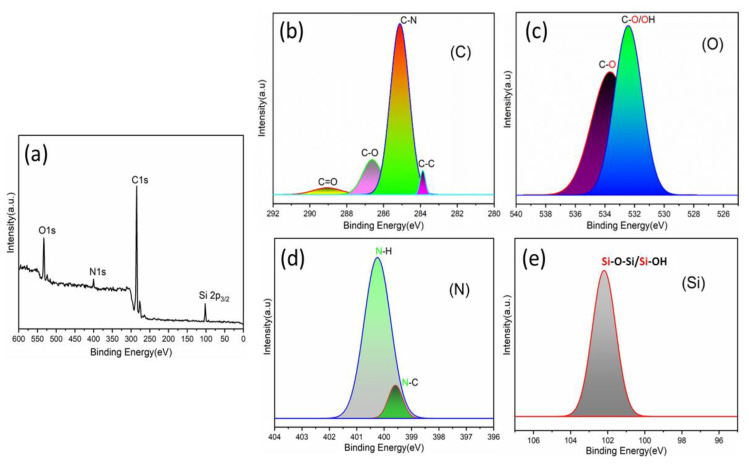
XPS analysis of LFAOIH@GO_0.5_−PU_35,_ (**a**) Survey spectrum, (**b**) C 1s peaks, (**c**) O1s peaks and (**d**) N1s 1s peaks and (**e**) Si 2p.

The chemical composition of LFAOIH@GO_0.5_-PU_35_ was thoroughly investigated using XPS analysis (Figure 3). The resulting deconvoluted spectra, depicted in Figure 3 for their respective elements (C 1s, N 1s, O 1s, and Si 2p_3/2_), offer detailed insights into the material’s composition. In the deconvoluted C1s spectrum, distinctive peaks were identified at four different binding energies: 283.6 eV, 285.2 eV, 286.5 eV, and 289 eV [23]. These peaks are associated with specific carbon bonding configurations, namely C-C, C-N, C-O, and C=O bonds, respectively [23]. Within the deconvoluted N1s spectrum, two prominent peaks were discerned at 399.6 eV and 400.25 eV, revealing the presence of nitrogen atoms engaged in N-C and N-H bonds [24]. In the deconvoluted O1s spectrum, two well-defined peaks were observed at 532.5 eV and 533.6 eV, indicating the binding energies of oxygen atoms in C-O/C-OH and C-O bonds [24]. Additionally, the characteristic Si 2p3/2 peak at 102.1 eV, as observed, signifies the existence of Si-O-Si and Si-OH bonds within the analyzed material [25]. This XPS analysis provides valuable insights into the chemical composition of LFAOIH@GO_0.5_-PU_35_, revealing the nature of carbon, nitrogen, oxygen, and silicon bonds present within the material.

#### 3.1.4. Physicomechanical Properties

Table 1 presents the physicomechanical impacts from LFAOIH-PU_35_ to LFAOIH@GO_X_-PU_35_. The study contrasts the physical and mechanical attributes of GO-infused PU with those of pure PU. The results indicate that the inclusion of GO enhances the scratch hardness, pencil hardness, cross hatch and gloss values as observed from LFAOIH-PU_35_ to LFAOIH@GO_X_-PU_35_. The enhancement in mechanical properties is also correlated to the presence of hydrogen bonding that is confirmed by the FTIR analysis of the samples. This improvement can be attributed to effective crosslinking through them [5]. Notably, the cross-hatch adhesion findings substantiate heightened adherence to mild steel strips.

Also, the incorporation of GO positively influences the satisfactory results observed in the physico-mechanical tests of all coating systems. This enhancement may be attributed to the presence of functional groups such as −OH and >C=O, along with double bonds (the saturation levels can differ based on the fatty acid composition found in the parent seed oil) and extended hydrocarbon chains [14,21]. These constituents contribute to the plasticizing effect on the coatings, thereby ensuring enhanced flexibility. However, LFAOIH@GO_0.5_-PU_35_ demonstrates brittleness during bend tests as the value of GO also affects the impact resistance, gloss values and cross hatch and other values decrease as GO value increases as shown in Table 1. It may be because the loading of GO increases; these attributes exhibit a declining trend [21,26]. Elevated GO concentrations lead to the formation of agglomerates, resulting in adversely affecting the overall coating performance [21]. Hence, out of all, LFAOIH@GO_0.5_-PU_35_ showed the best physicomechanical results and was optimized for further studies.

#### 3.1.5. XRD

**Figure 4:**
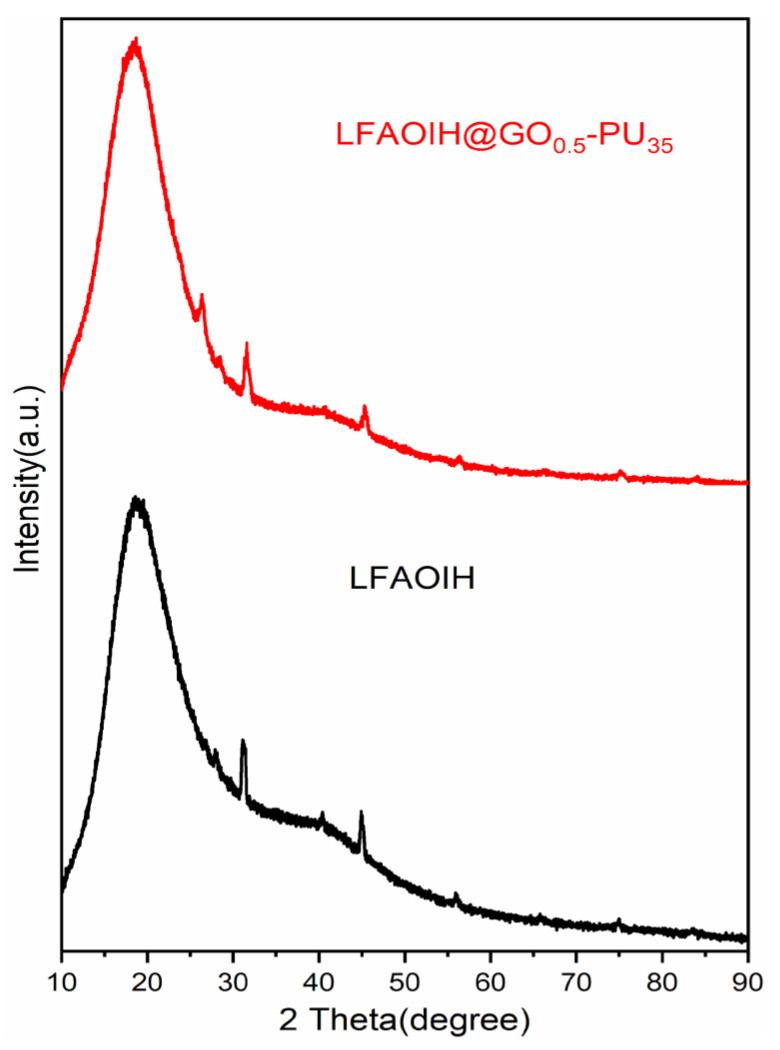
XRD thermograms of LFAOIH and LFAOIH@GO_0.5_-PU_35_.

XRD analyses were performed on both LFAOIH-PU_35_ and the doped variant with GO LFAOIH@GO_0.5_-PU_35_ to investigate possible modifications in the crystalline characteristics of the LFAOIH-PU_35_ matrix after introducing inorganic components and displayed in Figure 4. The XRD profiles of the materials demonstrated a consistent characteristic pattern, featuring a broad hump at 2θ = 21°, indicative of the amorphous nature of the matrix for both LFAOIH-PU_35_ and LFAOIH@GO_0.5_-PU_35_ due to the presence of silicon. Notably, smaller peaks were also observed around 32° and 45°. Upon doping with GO, the diffraction pattern exhibited similarities, with an additional peak emerging at 26° [27]. This emergence could potentially be attributed to the presence of graphite within the polymer matrix. Overall, both the LFAOIH-PU_35_ to LFAOIH@GO_0.5_-PU_35_ showed a semicrystalline behavior as shown in Figure 4.

#### 3.1.6. Thermal Analysis

**Figure 5:**
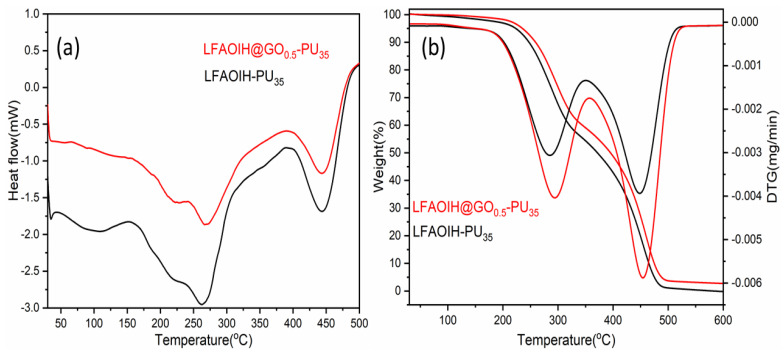
(**a**) DSC and (**b**) TGA/DTG thermograms of LFAOIH and LFAOIH@GO_0.5_-PU_35_.

The DSC thermogram analysis revealed the presence of two distinct endothermic transitions (Figure 5a). For LFAOIH-PU_35_, the initial endotherm occurs in the temperature range of 239 to 280 °C, with a peak centered at 263 °C. The subsequent endotherm takes place between 400 and 475 °C, with its peak at 438 °C. In the case of the LFAOIH@GO_0.5_-PU_35_ nanocomposite, the first endotherm transpires from 240 to 312 °C and is centered at 270 °C, while the second endotherm spans the range of 401 to 477 °C, with a peak at 440 °C. The first endothermic transition is associated with a phase transition, while the second is attributed to melting phenomena [14]. The observed shifts in these endotherms signify enhanced thermal properties brought about by the incorporation of GO. These shifts underline the improvement in thermal stability of the LFAOIH@GO_0.5_-PU_35_ matrix through the incorporation of GO, indicative of effective intermolecular interactions within the structure [21].

The TGA thermograms of LFAOIH-PU_35_ and LFAOIH@GO_0.5_-PU_35_ exhibit a biphasic degradation pattern, which is also evident in the corresponding DTG thermogram (Figure 5b). Up to 225 °C and 235 °C for LFAOIH-PU_35_ and LFAOIH@GO_0.5_-PU_35_, respectively, a mass loss of 5 wt% is observed due to the evaporation of moisture and volatile impurities. Subsequently, at 475 °C and 480 °C, a significant 90 wt% loss signifies the comprehensive degradation of the oil component, marked by the breakdown of the ester, amide and hydrocarbon chain in LFAOIH-PU_35_ and LFAOIH@GO_0.5_-PU_35,_ respectively [14]. These degradation trends are supported by the presence of two endothermic peaks in the DTG thermograms, observed at 275 and 445 °C for LFAOIH-PU_35_ and at 300 and 455 °C for LFAOIH@GO_0.5_-PU_35_ [14]. This improved stability is attributed to the effective dispersion of GO within the matrix, promoting favorable interactions that contribute to the enhanced thermal properties [21].

#### 3.1.7. Contact Angle

**Figure 6:**
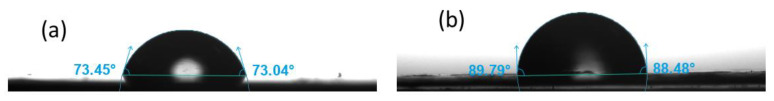
Contact angle analysis of (**a**) LFAOIH and (**b**) LFAOIH@GO_0.5_-PU_35_.

Surface wettability was assessed through contact angle (CA) studies, revealing the hydrophilic or hydrophobic nature of the OIH coating. The CA signifies the molecular interaction strength between liquids and solids. By depositing a 0.1 mL double-distilled water droplet onto the pristine interfaces of LFAOIH-PU_35_ and LFAOIH@GO_0.5_-PU_35_ the CA was measured. The outcomes of CA measurements (as depicted in Figure 6) unveiled a rise in the contact angle 73.25 G.U (73.45°–73.04°) in LFAOIH-PU_35_ to 88.64 G.U (88.79°–88.48°) in LFAOIH@GO_0.5_-PU_35_. This shift indicates a subtle enhancement in hydrophobicity following the incorporation of GO [21]. The observed augmentation in hydrophobicity could be attributed to the presence of hydrocarbon chains within the IOH coating [28]. This enhancement in the contact angle value is unequivocally linked to the integration of GO, fostering the development of a nanostructured assembly that augments surface roughness π -π stacking interactions, and the existence of hydrophobic domains. Consequently, such structural enhancement offers promising prospects for leveraging these coatings in humid environments [29].

#### 3.1.8. Morphology

**Figure 7:**
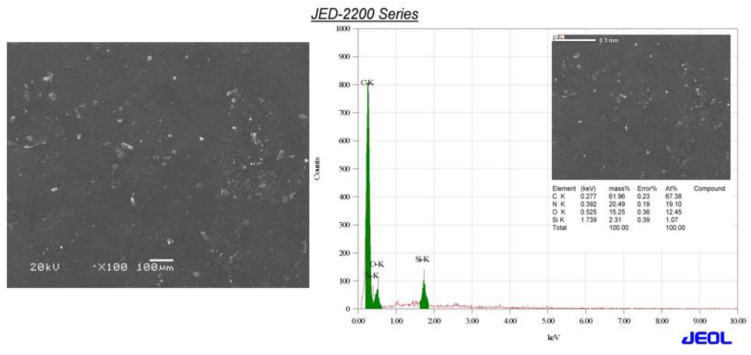
SEM and EDX micrograph of LFAOIH@GO_0.5_-PU_35_.

**SEM:** SEM analysis was systematically executed on LFAOIH@GO_0.5_-PU_35_ in order to comprehensively evaluate its surface morphology and assess the consequential effects stemming from the dispersion of GO within the OIH matrix (Figure 7) [21]. The analytical results unveiled a discernible roughness on the surface, which was attributed to the precise incorporation of GO at the nanoscale. Furthermore, the analysis detected the presence of rod-like or spherical-shaped features, augmenting the overall textural roughness of the surface. The identification of Si is discernible from the observed peaks at 1.7 KeV. The EDX spectra signify the existence of several elements, encompassing C, N, O, and Si. The absence of any other elements further suggests the purity of the polymer matrix.

**Figure 8:**
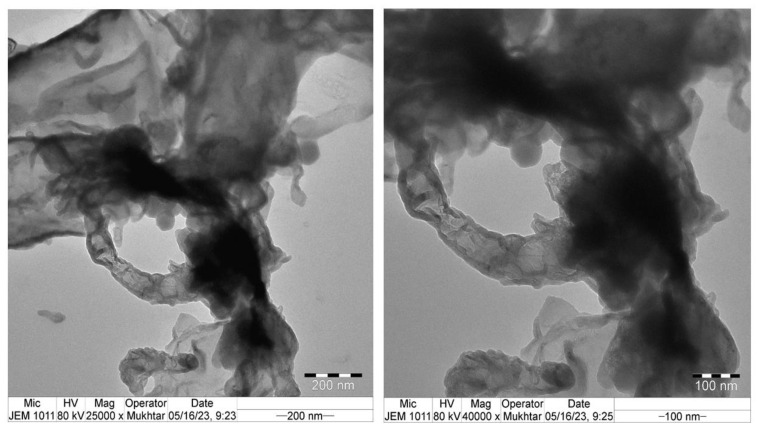
TEM micrograph of LFAOIH@GO_0.5_-PU_35_ at 100 nm and 200 nm.

**TEM:** TEM studies have elucidated a conspicuous aggregation of particulate material, distinctly distributed across the surface (Figure 8). The densely concentrated dark regions observed in the micrograph are indicative of the uniform dispersion of GO nanoparticles within the LFAOIH@GO_0.5_-PU_35_ matrix [21]. These particulates represent these entities generated through the hydrolytic condensation of TEOS. This phenomenon suggests the occurrence of SiO_2_ grafting onto the surface of GO [30].

#### 3.1.9. Anticorrosion Study

Electrochemical impedance spectroscopic (EIS) analysis

**Figure 9:**
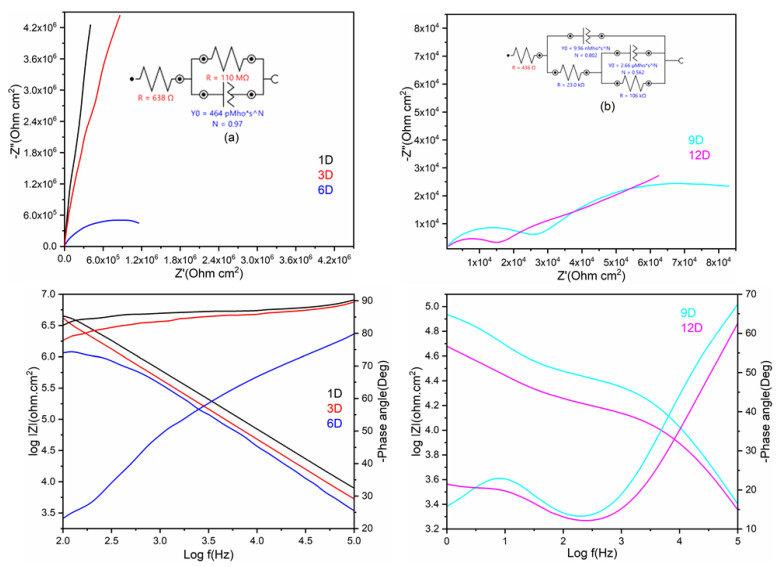
EIS and Bode theta analysis for LFAOIH-PU_35_ for various (1, 3, 9, 12) days.

**Figure 10:**
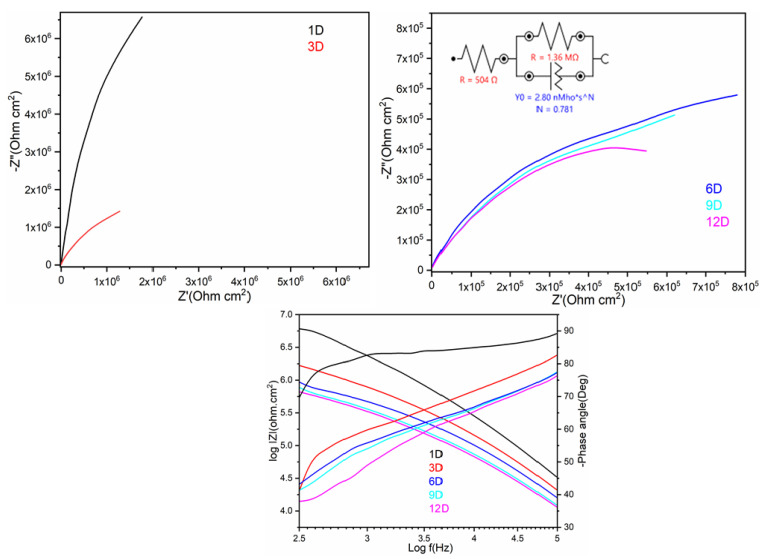
EIS and Bode theta analysis for LFAOIH@GO_0.5_-PU_35_ for various (1, 3, 9, 12) days.

To assess the anti-corrosive performance of coatings comprising LFAOIH-PU_35_ and LFAOIH@GO_0.5_-PU_35_ on mild steel strips (measuring 1.0 cm^2^), EIS measures have been conducted at various time intervals (1, 3, 6, 9, and 12 days) subsequent to exposure to a 3.5% NaCl environment. Figure 9 and Figure 10 present the Nyquist plots for LFAOIH-PU_35_ and LFAOIH@GO_0.5_-PU_35_ over a 12-day exposure period. In the analysis of these Nyquist plots, diverse equivalent circuit models are employed to effectively fit the experimental data. These Nyquist plots serve as valuable tools for unraveling the intricate interactions transpiring at the interface between the metallic substrate and the surrounding solution. They enable us to discern the onset of corrosion, the ingress of water through the coating-metal interface, and the subsequent formation of corrosion byproducts [9]. Furthermore, they facilitate the determination of critical parameters, including solution resistance (Rs), coating resistance (Rc comprising Rp1 and Rp2), and open circuit potential (OCP), as presented in the accompanying Table 2.

To gain a more comprehensive understanding of the interaction transpiring at the metal–solution interface, it is imperative to consider the values associated with the constant phase elements (CPE_1_ and CPE_2_). The CPE serves as an indicator of the extent to which practical behavior deviates from the ideal capacitive behavior within the framework of the equivalent circuit model. Its inclusion within this model is intended to mitigate systematic errors, ultimately contributing to the precision of the fitting results. The CPE is characterized by an exponent denoted as “n” (0 ≤ n ≤ 1), which quantifies the degree of deviation from ideal dielectric behavior. For LFAOIH-PU_35_, CPE_1_ signifies a typical double-layered capacitor, characterized by significant porosity, as indicated by its n_1_ values approximating 1 (0.97), while CPE_2_ exhibits Warburg-like attributes, with n_2_ values hovering around 0.56. This suggests a notable level of corrosion resistance for the surface of the manufactured coatings. Conversely, for LFAOIH@GO_0.5_-PU_35_, a single circuit with a CPE value within the range of 0.781 is observed at the 12th day of exposure, indicating that the dispersion of GO contributes to enhanced corrosion resistance [21]. Rc values, directly linked to the anti-corrosion effectiveness of coatings, reveal higher values associated with superior corrosion resistance. Notably, Rp1 represents the charge transfer resistance at the interface between LFAOIH-PU_35_ coatings and the solution, while Rp2 denotes the polarisation resistance at the interface between a corrosion product layer and the solution [21]. The overall polarisation resistance is a result of the parallel combination of Rp1 and Rp2. Conversely, Rs, signifying solution resistance, demonstrates a gradual decline as the exposure period lengthens. This suggests that, over time, the surface integrity of the coated mild steel strips weakens, enabling the easier diffusion of corrosive ions. In cases where coatings lack corrosion resistance, a substantial and rapid decrease in Rs (solution resistance) may occur over time. However, in the present study, we observe a gradual reduction in Rs, with the minimum value reached after a 12 day exposure period. Notably, the data reveal that, even after 12 days of exposure, LFAOIH@GO_0.5_-PU_35_ exhibits a higher Rs value compared to LFAOIH-PU_35_, highlighting the role of GO in providing an effective barrier against the diffusion of corrosive ions and preserving the integrity of the underlying metal surface [21]. Additionally, we observe that the Open Circuit Potential (OCP) values for LFAOIH@GO_0.5_-PU_35_ are initially higher (more positive) on the first day of immersion, but they progressively become more negative as the exposure time lengthens, especially when compared to LFAOIH-PU_35_. This behavior underscores the dynamic response of LFAOIH@GO_0.5_-PU_35_ to the corrosive environment over time.

The impedance behavior, following different immersion durations, is visually represented in Figure 10 through a Nyquist and Bode plot. The Nyquist plot displays the real impedance against imaginary impedance for pure LFAOIH-PU_35_ and GO dispersed LFAOIH@GO_0.5_-PU_35_ after 1, 3, 6, 9, and 12 days of immersion. The corrosion rate is discernible from the size of the Nyquist plot, where a larger diameter corresponds to a slower corrosion rate or a heightened resistance to corrosive ions [31]. In the case of both LFAOIH-PU_35_ and LFAOIH@GO_0.5_-PU_35_ coatings, an initial phase featuring a linear trend was noted on the first day of exposure. This occurrence can be attributed to the fact that not all organic coatings exhibit long-term impermeability. Consequently, ions and water have the potential to infiltrate through the capillary channels and pores inherent in the coating structure, leading to a reduction in resistance (Rc). With prolonged exposure of a coated surface to a corrosive environment, any voids or capillary channels within the coating permit the ingress of water molecules and corrosive ions through the coating–metal interface. This ingress subsequently results in a reduction in resistance over extended periods of immersion. Notably, an arc emerges at the initiation of the first semicircle in the EIS spectrum after 3 days of submersion for both LFAOIH-PU_35_ and LFAOIH@GO_0.5_-PU_35_ coatings [9]. Furthermore, it is worth emphasizing that the radii of these semicircles exhibit a gradual reduction over time. This trend indicates a potential acceleration in the corrosion rate, which can be attributed to various factors such as the formation of fresh coating pores, an expansion in the exposed surface area of pre-existing pores, or the existence of structural irregularities and imperfections within the coating matrix. After 9 days of exposure, a semicircle with a straight line was observed for LFAOIH-PU_35_, which indicated a combination of two impedance components. The semicircle represents a capacitive or diffusive process, while the straight line corresponds to a purely resistive process. This combination often indicates a complex electrochemical behavior at the studied interface. The semicircle portion may be associated with charge transfer resistance or the presence of a protective barrier, while the straight line suggests a more simplistic resistive behavior, possibly related to ionic conduction or diffusion through the coating with the value of 6 × 10^4^ ohm-cm^2^. Whereas, for LFAOIH@GO_0.5_-PU_35_, the arc of the semicircle has been decreased, which is found in the range of 6 × 10^5^ ohm-cm^2^, which is higher as compared to the case of LFAOIH-PU_35_.

Moreover, the assessment of corrosion resistance was corroborated through the utilization of a Bode plot (Figure 9). Bode impedance and phase angle graphs were generated as a function of frequency subsequent to the immersion of the coatings in 3.5% NaCl solutions. The logarithm of impedance (Log Z) exhibits a consistent monotonic decrease across all frequencies as the coatings undergo immersion, indicative of capacitive behavior for both LFAOIH-PU_35_ and LFAOIH@GO_0.5_-PU_35_ [9,32]. The impedance modulus (|Z| value), which serves as a semiquantitative indicator of the coating’s potential as a barrier, exhibits a direct correlation with the coating’s resistance and capacitance. Figure 10 illustrates higher phase angles (37°) and elevated impedance values than LFAOIH@GO_0.5_-PU_35_, persisting even after exposure over 12 days. These observations show that both the coatings show good accounts for corrosion resistive behavior, but LFAOIH@GO_0.5_-PU_35_ has higher anticorrosion properties, reinforcing its efficacy in mitigating corrosion.

Tafel Analysis

**Figure 11:**
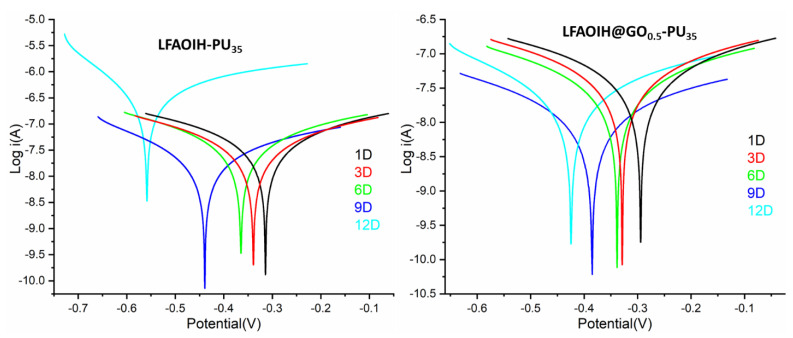
Tafel analysis for LFAOIH-PU_35_ and LFAOIH@GO_0.5_-PU_35_ for various (1, 3, 9, 12) days.

Tafel slopes are determined by extrapolating data from the Tafel region within polarization curves to examine whether the coatings are cathodic or anodic or both (Figure 11). A Tafel plot is employed to visually examine electrochemical characteristics, portraying the *X*-axis as potential and the *Y*-axis as the logarithm of current density. As evident in Figure 11, treating a mild steel substrate with LFAOIH-PU_35_ and LFAOIH@GO_0.5_-PU_35_ coatings leads to a shift in both the anodic and cathodic branches towards lower current densities showing the corrosion inhibition. In Table 3, we present critical electrochemical parameters for LFAOIH-PU_35_ and LFAOIH@GO_0.5_-PU_35_, E_corr_, I_corr_, corrosion rate, Open Circuit Potential (OCP), and IE values. E_corr_ and I_corr_ are interconnected through fundamental electrochemical principles, enabling the assessment and comparison of corrosion resistance in diverse materials and environments. A more positive E_corr_ signifies superior corrosion resistance, while a lower I_corr_ value suggests enhanced corrosion resistance. Analysis of Table 3 reveals that the E_corr_ value for LFAOIH@GO_0.5_-PU_35_ remains more positive (−0.424 > −0.557) in comparison to LFAOIH-PU_35_, even after 12 days. Conversely, Icorr values increase for both materials with prolonged exposure. The decrease in IE% over time is minimal, even after 12 days of immersion. These findings underscore the superior corrosion resistance of LFAOIH@GO_0.5_-PU_35_ nanocomposites over LFAOIH-PU_35_. This enhanced corrosion resistance is attributed to the incorporation of GO, which significantly augments the barrier effect, thereby suppressing the rate of the anodic reaction (primarily the oxidation of iron) and retarding the corrosion process. Consequently, LFAOIH@GO_0.5_-PU_35_ exhibits notable anticorrosive properties as a result of these aforementioned mechanisms.

## 4. Conclusions

An environmentally friendly and efficient synthesis method has been developed for the production of LFAOIH@GO_0.5_-PU_35_ using *Leucaena leucocephala,* a bio-resource. This approach involves the synthesis of LFAOIH-PU with 30–40% IPDI and the addition of a small quantity (0.25–0.75 wt%) of GO to create an LFAOIH@GO_0.5_-PU_35_ nanocomposite. The formulations selected for further investigation were LFAOIH-PU_35_ and LFAOIH@GO_0.5_-PU_35_. The chemical structure of these nanocomposites was elucidated using various analytical techniques, including FTIR, ^1^H, ^13^C, ^29^Si NMR, and XPS analysis. The study comprehensively explored the influence of different GO loadings on the nanocomposites structural, morphological, thermal stability, mechanical properties, and corrosion resistance. Several mechanical properties, including scratch hardness, pencil hardness, impact resistance, bending, gloss value, crosshatch adhesion, and thickness, were meticulously examined. Morphological characteristics were investigated through XRD, FE-SEM, EDX, and TEM analyses, revealing distinct surface roughness features. Remarkably, the resulting nanocomposites, particularly LFAOIH@GO_0.5_-PU_35_, displayed substantial improvements in mechanical properties, including significantly enhanced scratch hardness (3 Kg), pencil hardness (5H), and increased gloss values (79). Moreover, these nanocomposites exhibited exceptional thermal stability, retaining their structural integrity at temperatures up to 480 °C. Additionally, they demonstrated enhanced surface hydrophobicity, as indicated by a contact angle of 88.79°/88.48°, achieved with a minimal (0.50%) loading of GO. Crucially, EIS studies, including Nyquist, Bode, and Tafel plots, provided compelling evidence of the outstanding anti-corrosion performance of these nanocomposites. Consequently, these studied nanocomposites hold significant promise for the large-scale production of robust anticorrosive coatings with enhanced mechanical properties and durability.

The future competitiveness of OIH nanocomposite anticorrosive coatings is contingent on their capacity to address the mounting demand for corrosion protection in diverse industrial sectors. Ongoing innovations in nanotechnology hold the potential to enhance performance through the development of advanced nanomaterials. The ability to tailor these coatings to meet the specific corrosion challenges of various industries, coupled with their long-term durability and cost-effectiveness, will be pivotal for maintaining a competitive edge. These coatings are well-positioned to not only provide corrosion resistance, but can also offer supplementary functionalities such as self-healing and antimicrobial properties, rendering them multifaceted. Furthermore, ensuring compatibility with emerging materials and substrates, expanding their global reach, substantial investments in research and development, and fostering collaboration with scientists and industry stakeholders are integral to their sustained competitiveness within a dynamic and burgeoning market.

## Figures and Tables

**Table 1:** Various mechanical properties of LFAOIH-PU_35_ and LFAOIH@GO_x_-PU_35_.

Properties	LFAOIH-PU_35_	LFAOIH@GO_0.25_-PU_35_	LFAOIH@GO_0.5_-PU_35_	LFAOIH@GO_0.75_-PU_35_
Scratch hardness (kg)	2	2.4	3.0	2.8
Impact (lb/inch) 150	Pass	Pass	Pass	fail
Bending (1/8)	pass	pass	pass	pass
Pencil hardness	3H	4H	5H	4H
Cross Hatch (%)	100	100	100	98
Gloss at 60°	69	75	79	82
Thickness (micron)	88	129	135	140

**Table 2:** EIS studies of LFAOIH-PU_35_ and LFAOIH@GO_0.5_-PU_35_ coatings.

**LFAOIH-PU_35_**										
Time (Day)		OCP (V)	χ^2^	Rs (Ω/cm^2^)	Rc (MΩ/m^2^)	CPE_1_				
						Y_0_, pMho*s^n^	n			
1		0.183	0.090	638	110	464	0.97			
3		−0.281	0.243	622	58	443	0.95			
6		−0.409	0.114	615	25	391	0.79		CPE_2_	
					Rp1 (KΩ/cm^2^)	Y_0_, nMho*s^n^	n_1_	Rp2 (KΩ/cm^2^_)_	Y_0_, µMho*s^n^	n_2_
9		−0.473	0.078	436	23	9.96	0.80	106	2.66	0.56
12		−0.478	0.074	425	11.1	10.2	0.79	297	12.4	0.365
**LFAOIH@GO_0.5_-PU_35_**										
Time (Day)	OCP (V)		χ^2^	Rs (Ω/cm^2^)	Rc (MΩ/cm^2^)	CPE				
						Y_0_, nMho*s^n^			n	
1	0.211		0.708	616	53.4	3.58			0.911	
3	−0.311		0.192	572	14.5	3.43			0.801	
6	−0.325		0.157	527	12.5	3.67			0.782	
9	−0.356		0.134	526	1.50	2.97			0.782	
12	−0.402		0.152	504	1.36	2.80			0.781	

**Table 3:** Corrosion Potentiodynamic of LFAOIH-PU_35_ and LFAOIH@GO_0.5_-PU_35_ coatings.

Immersion Time (Day)	E_corr_ (V)	i_corr_ (A/cm^2^)	Corrosion Rate (mm/Year)	LPR (Ω)	OCP(V)	IE(%)
LFAOIH-PU_35_						
1	−0.314	1.282 × 10^−8^	2.430 × 10^−4^	3.895 × 10^6^	−0.311	99.97
3	−0.338	2.100 × 10^−8^	2.689 × 10^−4^	2.745 × 10^6^	−0.333	99.95
6	−0.364	2.314 × 10^−8^	2.930 × 10^−4^	2.683 × 10^6^	−0.355	99.94
9	−0.440	2.521 × 10^−8^	3.091 × 10^−4^	2.304 × 10^6^	−0.410	99.94
12	−0.557	1.591 × 10^−7^	1.845 × 10^−3^	1.928 × 10^5^	−0.478	99.96
LFAOIH@GO_0.5_-PU_35_						
1	−0.303	2.170 × 10^−8^	2.249 × 10^−4^	3.846 × 10^6^	−0.300	99.95
3	−0.328	2.780 × 10^−8^	2.521 × 10^−4^	2.501 × 10^6^	−0.325	99.34
6	−0.338	2.936 × 10^−8^	3.245 × 10^−4^	2.315 × 10^6^	−0.333	99.93
9	−0.385	2.350 × 10^−8^	5.556 × 10^−4^	2.024 × 10^6^	−0.382	99.94
12	−0.424	7.016 × 10^−7^	8.153 × 10^−3^	7.909 × 10^5^	−0.402	99.83

## Data Availability

The data are available from the corresponding author on reasonable request.
